# Genome sequence of the yeast *Candida solani* UCD2211 isolated from soil in Ireland

**DOI:** 10.1128/mra.01409-25

**Published:** 2026-02-09

**Authors:** Padraic G. Heneghan, Róisín O’Neill, Uliana Osinovskaia, Adam P. Ryan, Edvin Sebastian, Fiona Silitrari-Chen, Alexander Vial, Isabella Vozza, Vincent Walsh Giguere, Tadhg Ó. Cróinín, Kenneth H. Wolfe, Geraldine Butler, Kevin P. Byrne

**Affiliations:** 1 School of Medicine, Conway Institute, University College Dublin 37438, Dublin, Ireland; 2 School of Biomolecular and Biomedical Science, Conway Institute, University College Dublin 206442, Dublin, Ireland; University of California Riverside, Riverside, California, USA

**Keywords:** yeasts, genome analysis

## Abstract

*Candida solani* is a member of the *Wickerhamomyces* clade of budding yeasts. We present the genome sequence of *C. solani* strain UCD2211 isolated from soil on the University College Dublin (UCD) campus in Ireland. This genome was 12.67 Mb and assembled into six chromosome-sized contigs plus a mitochondrial genome contig.

## ANNOUNCEMENT

*Candida solani* is an asexual species that is a member of the *Wickerhamomyces* clade of budding yeasts (1). It was first isolated from a potato-starch mill in the Netherlands (2). It has no known food or biotechnological applications. As it does not grow above 35°C, it is unlikely to have pathogenic capacity in humans. Two other genome assemblies are available: from type strain NRRL Y-2224 fragmented in over 9,000 contigs (1) and a chromosome-level assembly from a related strain UCD1087 (3).

*C. solani* UCD2211 was isolated from soil collected on the University College Dublin (UCD) Campus in Dublin, Ireland (GPS coordinates 53.308583, −6.222999) as part of an undergraduate research module (4). Soil material was passaged twice at room temperature in 9 mL liquid yeast extract-peptone-dextrose (YPD) containing chloramphenicol (30 μg/mL) and ampicillin (100 μg/mL) and cultured on YPD agar plates. The species was identified by ribosomal DNA as previously described (3). Sequence identity was 96.98% (546/563 bp) in the ITS and 99.46% (556/559 bp) in the D1/D2 region to the type strain of *C. solani* (accession numbers KY102402 and NG_055191).

DNA was isolated by phenol/chloroform extraction from liquid YPD cultures grown at room temperature. Short-read sequencing using a NovaSeq X Plus (Novogene [UK] Company, Ltd.) yielded 17.6 million read pairs (2 × 150 bp). Adapters and low-quality reads were removed (Skewer v0.2.2) (5). For long-read sequencing, after DNA extraction (MasterPure Yeast DNA Purification Kit MPY80010) and library preparation (Native Barcoding Kit SQK-NBD114-24), we selected for DNA >3 kb with long fragment buffer before Oxford Nanopore sequencing (MinION MK1B, flowcell FLO-MIN114), and super accurate basecalling (MinKNOW v24.06.16; default demultiplexing; barcode trimming; reads ≥ 500 bp kept; no quality cut-off). NanoFilt (v2.3.0) (6) retained 184,681 reads with quality ≥10 and length ≥1,000 bp (reads N50 = 6,932 bp), which were then assembled using Canu (v2.2) (7), followed by five rounds of error correction using NextPolish (v.1.4.1) with the Illumina reads (8). Default parameters were used, except where otherwise noted.

The assembly consists of six nuclear contigs (total 12.63 Mb; N50 = 2,190,064 bp) and the mitochondrial genome (38,626 bp circular unit; accession number CDRNKE010000007). All six nuclear contigs appear to be complete chromosomes because every end of every contig is either a probable telomere repeat sequence (TGGTGTAGGGATGTACTGTG)_n_ (9) or an rDNA array (detected by BLASTN) (Table 1). All rDNA arrays are oriented with the 18S and 26S rRNA genes transcribed toward the nearby contig end. The genome is collinear with that of UCD1087 (3), except for a reciprocal translocation and an inversion (Table 1).

**TABLE 1:** Genome assembly characteristics of *Candida solani* UCD2211

Chromosome	Size (bp)	Left end	Right end	UCD1087 comparison
1	2,692,170	rDNA	rDNA	chr1 + part of chr4 (reciprocal translocation)
2	2,640,582	Telomere → rDNA	Telomere	chr4 + part of chr1 (reciprocal translocation)
3	2,190,064	rDNA	rDNA	chr2
4	2,155,114	rDNA	Telomere	chr3
5	1,523,230	Telomere	rDNA	chr6
6	1,426,753	Telomere	Telomere	chr5 (inversion on this chr)
mitochondrial	38,626	n/a (circular)	n/a (circular)	

The UCD2211 genome assembly has an average nucleotide identity (10) of 97.69% to the assembly of type strain NRRL Y-2224 (JAKVQS010000000) (1) and 93.2% to the assembly of UCD1087 (CAXWWC010000000), which we previously isolated (3), indicating the sequence diversity within *C. solani*. Using BUSCO v5.8.2 (11), genome completeness was estimated as 93.6% compared to the *Ascomycota* lineage data set. The guanine or sytosine (G + C) content was 39.86%.

## Data Availability

This Whole Genome Shotgun project has been deposited as DDBJ/ENA/GenBank accession number CDRNKE010000000 (BioProject no. PRJEB97773). The version described in this paper is version 1. The reads were deposited at ENA (accessions ERR15652871, ERR15761027). The ITS sequence is PX481655. The D1/D2 sequence is PX481656.
